# Using Cellulolytic Nitrogen Fixing Bacterium, *Azomonas agilis* for Effective Degradation of Agricultural Residues

**DOI:** 10.2174/1874285801812010154

**Published:** 2018-05-31

**Authors:** Zaw K. Latt, San S. Yu, Ei P. Kyaw, Tin M. Lynn, May T. Nwe, Wai W. Mon, Kyaw N. Aye

**Affiliations:** 1 Biotechnology Research Department, Ministry of Education, Kyaukse, Mandalay Division, 100301, Myanmar; 2 Department of Chemical Engineering, Yangon Technological University, Yangon, Myanmar

**Keywords:** *Azomonas agilis*, Nitrogen fixation, Cellulose, NaCl, Reducing sugar, Sustainable agriculture

## Abstract

**Introduction:**

*Azomonas agilis*, a nitrogen-fixing bacterium, was isolated from rhizospheric soil in central Myanmar.

**Methods & Materials:**

The nitrogen-fixing activity of this bacterium was detected by plate screening method using glucose nitrogen free mineral medium and ammonium test-kit Cellulolytic activity was screened by plat assay and detected by Dinitrosalicyclic acid method (DNS).

**Results & Discussion:**

The isolated *A. agilis* grew in media containing 3-12% of NaCl, although the growth became poor when NaCl concentrations increased. Among various carbon sources, sucrose was the best source for ammonium accumulation of this bacterium, whereas arabinose was not the suitable carbon source. Although the nitrogen-fixing activity of *A. agilis* was highest after one week incubation, cellulase enzyme production was highest after 2-3 days of incubation. It was observed that cellulase enzyme activity of *A. agilis* for cellulose and sodium carboxymethyl cellulose (CMC) was almost the same. Three agricultural wastes were used to detect the cellulase enzyme activity of *A. agilis*, cellulase activity was better on filter paper as a substrate when compared to rice-straw and sawdust.

**Conclusion:**

So, the isolated *A. agilis* has high potential as an effective bacterial strain to use in sustainable agriculture and degradation of some agricultural residues.

## 1 INTRODUCTION

In agriculture, the role of nitrogen is extremely important because proteins, nucleic acids and other essential molecules in all organisms constitute nitrogen. It is also an essential element for many biological processes, including those occurring in plants [1]. Although nitrogen is found abundantly in the atmospheric, nitrogen fertilizers have to be produced increasingly by the Harber–Bosch process annually due to the deficiency of ammonia produced by biological nitrogen fixation- the enzyme-catalyzed reduction of nitrogen gas (N_2_). The research is focused on nitrogen-fixing bacteria as there is concern over greenhouse gas emitted from the production of nitrogen fertilizer by the Harber-Bosch process, and in particular, genetic modification was made to excrete excess ammonia for agricultural purposes [2, 3].

Some important non-symbiotic nitrogen-fixing bacteria include *Achromobacter*, *Acetobacter*, *Alcaligenes*, *Arthrobacter*, *Azospirillum*, *Azotobacter*, *Azomonas*, *Bacillus*, *Beijerinckia*, *Clostridium*, *Corynebacterium*, *Derxia*, *Enterobacter*, *Herbaspirillum*, *Klebsiella*, *Pseudomonas*, *Rhodospirillum, Rhodopseudomonas* and *Xanthobacter* [4].

*Azotobacteriaceae* comprises two genera; non-cyst forming bacterial group; *Azomonas* with three species (*A. agilis*, *A. insignis* and *A. macrocytogenes*) and cyst-forming; *Azotobacter* comprising of 6 species namely, *A. chroococcum, A. vinelandii, A. beijerinckii, A. nigricans, A. armeniacus* and *A. paspali* [5].

*A. agilis* is Gram-negative bacteria and a species of motile. *A.agilis* is found in water and has the ability to fix atmospheric nitrogen. It is a typical strain for the genus *Azomonas*. Salt tolerance of this species is up to 1.0% and resistant to iodoacetate (1 μM). Therefore, it is suggested that it may have the ability to live in contaminated water with relatively high concentrations of organic matter and mineral salts [6]. In addition, *A.agilis* has contributed in the bioremediation of cadmium-polluted water [7].

Nowadays, although the sources of agricultural wastes are diverse, these solid wastes can be potential sources for hazardous and detrimental to the terrestrial and aquatic eco-systems. For the farmers, they have to manage the residues efficiently in time for subsequent year’s crop and also economically to save cost. It is widely seen that farmers usually burnt these residues in the field after harvest. Burning is not the most effective method for clearing of waste stubble from fields in preparation for the succeeding planting seasons. Uncontrolled and improper handling of agricultural wastes can often lead to many situations that will be concerned with an environmental issue. Of course, over-application of agricultural waste in the formed manure to cropland and pasture has a negative effect on crop production and can decrease in yields due to the effects of inhibitory amounts of ammonia and nitrite nitrogen (NO_2_-N) or salts in the soil farmers utilized. It was well documented that excess loading of nitrogen and phosphorus from using agricultural waste to land on eutrophication of water bodies or contamination of drinking water [8-10].

There are many research papers that are well documented for the nitrogen-fixing activity of *Azotobacter* spp., *Azospirillum* spp., and *Rhizobium* spp. However, the nitrogen-fixing activity of *Azomonas* spp. and their characteristics have not been well documented, and few papers give information about *Azomonas* spp.

In Myanmar, an agricultural country, most farmers grow rice as one of the major crops. Especially, the Irrawaddy region of Myanmar is the major rice-growing regions area of the country. Rice growing area of this region is affected by salinity. The salinity level is increased yearly. The effects of soil salinity on agricultural yield is enormous as the establishment, growth and development of plants are affected that leads to huge losses in productivity [11]. It was also reported that some of the microorganisms, particularly valuable bacteria and fungi can help to develop plant performance under stress condition and, improve yield [12].

After harvesting crops in fields, many agricultural wastes have been left. The burning of these wastes caused air pollution and other problems. For degradation of these wastes into valuable products, it is needed to have cellulase-producing microorganisms in soils. Moreover, it will be expected to develop nitrogen-rich products from degradation of these wastes as biofertilizer. Therefore, this research aims to obtain a good candidate strain for cellulolytic nitrogen-fixing activities and Myanmar’s agriculture.

The objectives of this research work are to screen nitrogen-fixing activity in different conditions, some phenotypic characteristics, cellulase enzyme producing activity and utilization of some cellulosic wastes.

## 2 MATERIALS AND METHODS

### 2.1 Isolation of Bacteria

Soil samples were collected from different rhizospheric soils and under decayed rice straw in Kyaukse district and around Patheingyi Township, Mandalay Region, Myanmar. Glucose Nitrogen Free Mineral Medium (GNFMM) was used to isolate nitrogen-fixing bacteria. The composition of the isolated medium was as follows (g/L): 1.0 K_2_HPO_4_, 1.0 CaCl_2_, 0.5 NaCl, 0.25 MgSO_4_·7H_2_O, 0.01 FeSO_4_·7H_2_ or O, 0.01 Na_2_MoO_4_·2H_2_O, 0.01 MnSO_4_·5H_2_O and a carbon source was glucose (7 g/L). Solid medium was produced by adding 2% agar.

### 2.2 Screening of Nitrogen-Fixing Activity

The visual detection of nitrogen-fixing activity was observed by using NFMM with 0.7% Glucose. Bromothymol blue (BTB) was added to the media as an indicator. The media was prepared for both agar and broth. After 7 days incubation, changing the color of the medium was recorded. To detect nitrogen-fixing activity from the broth culture, the reagents of ammonium test kit (VISCOLOR Alpha Ammonium reagent, MACHEREY-NAGEL GmbH & Co. KG, Germany) which was kindly supported from Shibaura Institute of Technology, Japan, were added and the appeared color was noted by comparing the color chart on the test kit. There are three kinds of reagents of ammonium test kit. Two drops per 1 ml of ammonium test kit reagent (I) were added into the supernatant. And, one-fifth of reagent (II) was added and incubated for 5 minutes. Finally, one drop per 1 ml of reagent (III) was added and incubated again for 5 minutes. And then, the color development of supernatant was observed and compared with the color chart from the ammonium test kit.

### 2.3 Identification of Isolated Strain by 16S rDNA Sequencing

The best nitrogen-fixing strain was chosen and identified by 16S rDNA sequencing. DNA extraction was performed using the PrepMan reagent (Thermofisher Scientific, California, US) and primers 10F (5'-AGTTTGATCCTGGCTC-3') and 800R (5'-CTACCAGGGTATCTAAT-3'). 16S rDNA was amplified by PCR 30 cycles using a universal primer. Each cycle consisted of denaturation for 1 min at 94^°^C, annealing for 30 s at 56^°^C, and extension for 45 sec at 72^°^C and final extension for 45 sec at 72^°^C. Sequencing reactions were performed using the Big Dye Terminator Cycle Sequencing Kit (v.3.1) (Thermofisher Scientific, California, US) and analyzed in Genetic Analyzer 3130 sequencer (Applied Biosystems, US). Nucleotide sequences were analyzed using BLAST on the NCBI BLASTN and Greengenes.lbl.gov.

### 2.4 Characterization of Phenotypic Characteristics of Bacteria

Some phenotypic characteristics of *A. agilis* were studied and characterized such as Triple Sugar Iron Agar (TSI), Motility, Indole, Methyl Red, Citrate Utilization, Voges-proskauer, Starch hydrolysis, Gelatin liquification, Catalase, Cellulase, Urease, growth at different NaCl concentrations, some antibiotic sensitivity patterns and utilization of some sugars, glucose, sucrose, fructose, arabinose, cellulose, CMC (Carboxymethyl Cellulose), and mannitol.

### 2.5 Effects of Various Carbon Sources on Ammonia Accumulation

A single colony of the bacterium was inoculated into 10 ml of NFGMM broth and incubated at 37^°^C for 2-3 days before they were inoculated in nitrogen-free liquid medium with various carbon sources. 1ml of culture broth was inoculated into NFMM broth containing various carbon sources; glucose, sucrose, fructose, arabinose, cellulose, CMC and mannitol (7g/L for each sugar). After one week incubation, accumulated ammonium concentration in culture broth from various carbon sources was determined by Indophenol method [13]. Each experiment was replicated three times.

### 2.6 Effects of NaCl on Ammonia Accumulation

Various NaCl concentrations, 3%, 5%, 7%, 9% and 12% were used to screen the growth and ammonia accumulation of *A. agilis* using GNFMM. After inoculation in GNFMM broth containing various NaCl concentrations and incubation for one week, accumulated ammonium concentrations were measured by Indophenol method.

### 2.7 Cellulase Enzyme Assay

An inoculum from an overnight grown culture in log phase was added to 100 ml NFMM containing cellulose powder (Sigma-Aldrich) as a carbon source in 250-ml Erlenmeyer flasks. After incubation for 48 h, at 37^°^C, under shaking condition of 150 rpm, the culture was harvested and growth was measured at OD600 with UV-Vis spectrophotometer (Optima SP-3000 Plus, Slovakia). The cultures were centrifuged at 10,000 rpm for 10 min at 4^°^C. After centrifugation, the supernatant (cell-free extract) was used as crude preparation to measure cellulase activity.

For enzyme assay, Cellulose and Sodium CMC were used as the substrates. Enzyme activity was determined by measuring the release of reducing sugars during the enzyme-substrate reaction using dinitrosalicylic acid method [14]. The values were determined from glucose standard curve. One unit (IU) of activity was defined as the amount of enzyme required to liberate 1μmol of glucose per minute under given assay conditions.

### 2.8 Determination of theCellulolytic Activity of *A. agilis* Using Agricultural Residues

A single colony of the bacterium was inoculated into NFMM containing cellulose as a carbon source. After incubation at 37^°^C for 48 h, under shaking condition of 150 rpm, the supernatant (after centrifugation) was prepared for cellulase activity using agricultural residues. Different agro-residues like rice straw, filter paper and sawdust were used.

## 3 RESULTS AND DISCUSSION

### 3.1 Phenotypic Characteristics of the Isolated Strain

The isolated nitrogen-fixing bacterium was identified as *A. agilis* by 16S rDNA sequencing with 99.34% similarity. Colonial and microscopic morphology of *A. agilis* was shown in Fig. (**1**). Some phenotypic characteristics of *A. agilis* were characterized in (Table **1**).

### 3.2 Screening of Nitrogen-Fixing Activity

For preliminary screening of nitrogen-fixing activity by plate screening method, *A. agilis* caused color changing of the GNFMM from green to blue color after one-week incubation as shown in Fig. (**2**). Such kind of screening can be easily seen and select the bacteria having nitrogen fixing activity. Nitrogen-fixing bacteria that can excrete a significant amount of ammonia into their environment can change the color of the media. Nitrogen-fixing bacteria normally fixed atmospheric nitrogen and excreted ammonia from fixation into their surrounding environment. In this case, ammonia was accumulated in the media from the nitrogen-fixing activity of bacteria and increasing the ammonium concentration caused pH changing of the media. As a consequence, it caused color changing of the media. The finding was compatible with Gothwal *et al.*, Iwata *et al.* who described that nitrogen activity of *Azotobacter beijerinckii* and *Lysobacter enzymogenes* DMS 2043T and rhizospheric bacterial isolates were screened by changing the color of BTB containing nitrogen free media, and these strains showed a color change in BTB containing media, suggesting excretion of ammonia [15, 16].

**Bali *et al.*** found that the rise of pH to approximately 8.5 occurred in ammonia excretion of *Azotobacter vinelandii* for both wild type and mutant strains during the period when ammonium was released.

On detection with ammonia test kit, the supernatant of *A. agilis* culture broth gave color development after harvesting the bacterial broth. The estimated amount of above 3 ppm of ammonium concentration was obtained from the comparison of standard color chart on the test kit Fig. (**2**). This detection could give estimate amount of ammonium concentration. Ammonium concentration was increased after once week incubation. It was also reported that free-living diazotrophs fix dinitrogen sufficient for their own needs and do not generally excrete significant amounts of ammonium into their environment; fixed nitrogen is released after death and lysis of bacteria [17].

### 3.3 Effects of Various Carbon Sources on Ammonia Accumulation

*A. agilis* accumulated ammonia in NFMM broth in utilizing various carbon sources. Among seven carbon sources, the bacterium gave the highest amount of ammonium concentration 14.665 ppm from the utilization of sucrose as a carbon source and followed by fructose. It was found that ammonium concentration was the lowest in arabinose containing NFMM broth, which was only 0.326 ppm of ammonium concentration (Table **2**).

It was found that ammonia accumulation of *A.agilis* in NFMM broth was depended on carbon source. For significant ammonia excretion into their environment, it was needed that carbon source in the media should be their preference besides other physical parameters. Therefore, the nitrogen-fixing activity of *A.agilis* by using other nitrogen-free media should be studied. The dominant sugars in root exudates are glucose, fructose, galactose, arabinose, maltose, raffinose, rhamnose, sucrose and xylose [18]. Therefore, it can be assumed that isolated *A.agilis* can fix atmospheric nitrogen well and excrete ammonia into their environment when applied in field trial because sucrose is one of the dominant sugars in root exudates.

### 3.4 Effect of NaCl on Ammonia Accumulation

**Fig. (3):**
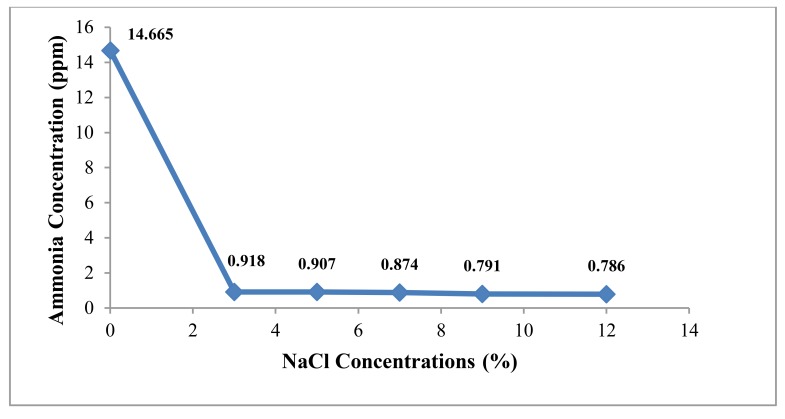
Accumulated ammonium concentrations of *A. agilis* in GNFMM broth with various NaCl concentrations. With increasing NaCl concentration, it was seen that ammonium concentration was obviously decreased.

*A.agilis* can grow on NFGMM with various NaCl concentrations in the range from 3% to 12% but growth became poor with increasing NaCl concentrations. In this study, it was obviously seen that NaCl effects on ammonia accumulation of *A. agilis* in the culture broth. Normally, NFMM contains 0.005% NaCl concentration and in this condition, the bacterium excreted ammonia concentration of about 14 ppm. When 3% NaCl concentration as the lowest and 12% as the highest concentration were used, the ammonia concentration in culture broth decreased from lowest concentration to highest concentration of NaCl. Ammonia concentration was obviously decreased when compared with the culture broth in original NFMM media (control). At the lowest concentration of NaCl, it was found that only 0.918 ppm of ammonium concentration could be accumulated. This concentration was very low and not significant excretion (Fig. **3**).

Nitrogen fixation is particularly sensitive to salt stress, the nitrogenase activity of *Klebsiella pneumoniae* was sharply decreased as soon as the osmolarity was increased, with no activity detected at NaCl concentrations higher than about 3% [19]. However, it was also stated that the nitrogen-fixing activity of salt-tolerant *Azotobacter* was maximal at 5–25% NaCl [20-22].

### 3.5 Cellulase Production in NFMM

Cellulolytic activity was assayed in nitrogen-free media because this research work aims to improve soil fertility by the nitrogen-fixing activity of diazotroph from the utilization of various agricultural residues. Cellulose and CMC in powder form of 0.2% were used as a substrate for assay. Reducing sugar concentrations were almost the same from both substrates as shown in Fig. (**4**). After 4 days incubation in broth culture, cellulase activity was decreased. Cellulase activity of *A.agilis* was not high because nitrogen deficient condition may inhibit *A. agilis* to produce cellulase enzyme. Higher production of enzyme requires the presence of complex nitrogen sources [23].

A maximum cellulose activity of 0.043 U/ml has been reported from cell-free culture supernatants of *Geobacillus* sp. isolated from a deep goldmine environment [24]. *Li *et al.** (2008) [25] reported maximum cellulase activity (0.26 U/ml) of a *Bacillus* sp. when the culture was grown in LB medium supplemented with 1% CMC. In another study, a cellulase activity of 0.0113 U/ml was observed under optimized conditions from *Geobacillus* sp [26].

### 3.6 Cellulolytic Activity of *A. agilis* Using Cellulosic Wastes

Among three different substrates, cellulase activity of *A. agilis* was higher in using filter paper and rice straw than the other one. Cellulase activity was less on Sawdust. It can be seen that the bacterium can easily degrade filter paper and rice straw than sawdust. It may promote the cellulase activity of the bacterium for the substrate of sawdust when nitrogen source into media was added. Results were shown in Fig. (**5**). Rice straw and filter paper are abundant in our country. After harvesting rice in the field, it is needed to effectively degrade rice straw. Utilization of cellulosic wastes as cheap carbon and energy sources can serve as an alternative to our rapidly depleting finite sources of fossil fuels.

## CONCLUSION

The nitrogen-fixing activity of *A. agilis* was obviously observed through screening and when detected by ammonium test kit and Indophenol method, accumulated ammonia concentration in culture broth was obtained. The bacterium can utilize various carbon sources but nitrogen-fixing activity was also depended on carbon sources. The highest amount of accumulated ammonia was detected in NFMM containing sucrose. NaCl also effects on growth and function of *A. agilis*. Its nitrogen-fixing activity was sensitive to the high concentration of NaCl. With increasing NaCl concentrations, nitrogen-fixing activity was decreased. In addition, *A. agilis* has cellulolytic activity when screened on NFMM containing cellulose and CMC. In quantitative determination, the cellulase of the bacterium can convert some cellulosic residues into reducing sugar. Among five substrates, reducing sugars converted from substrates were obtained. Therefore, the isolated *A. agilis* has high potential as an effective bacterial strain for agricultural application and degradation of some agricultural residues. However, it will not be suitable when this bacterium is applied in salt-affected agricultural soils. To be a good candidate strains for agricultural application, more repeated experiments and other important characteristics for agriculture should be studied.

## Figures and Tables

**Fig. (1):**
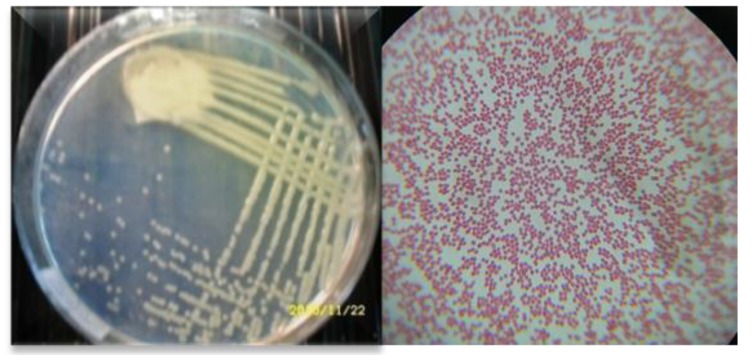
Colonial Morphology and Microscopic Morphology (x100) of *A. agilis*.

**Fig. (2):**
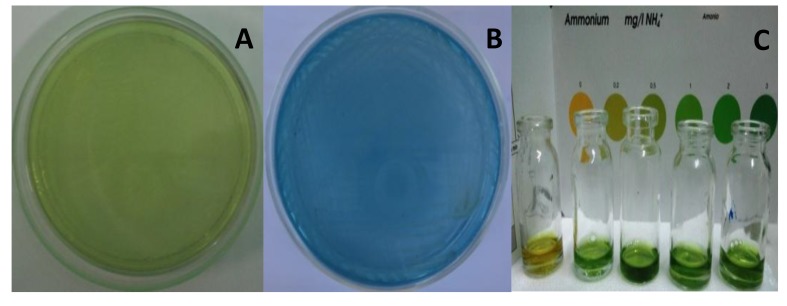
Screening on Nitrogen Fixing Activity, (a) Control, (b) Color Changing of Culture of *A. agilis* and (c) Detection by Ammonia Test Kit (the yellow bottle is the control).

**Fig. (4):**
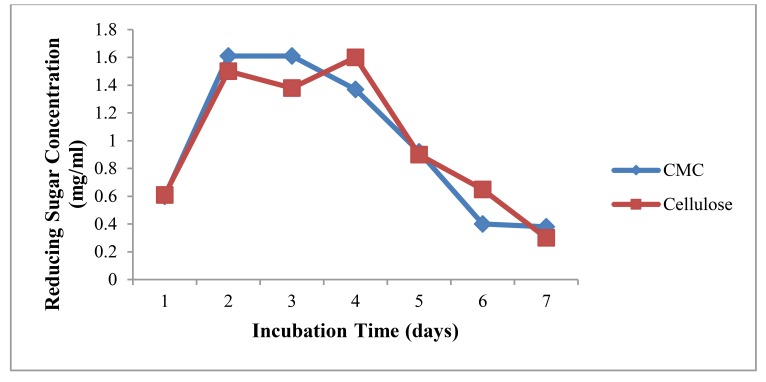
Reducing sugar concentrations converted from cellulose and CMC by cellulase activity of *A. agilis* in NFMM. This reducing sugar concentration was detected in nitrogen free condition for the bacterium. The concentrations were almost the same for both substrates.

**Fig. (5):**
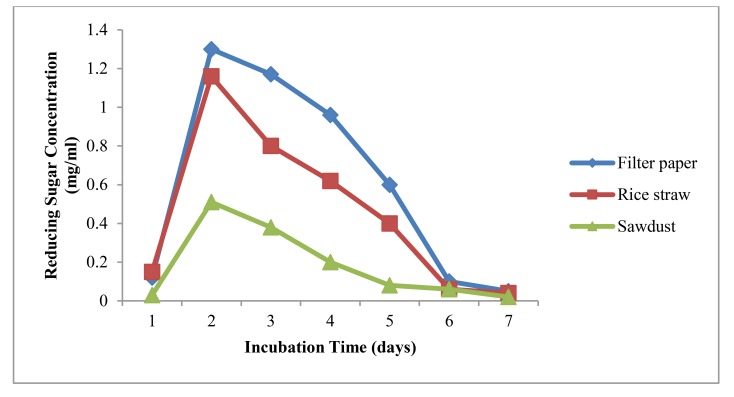
Reducing sugar concentrations converted from three substrates (filter paper, rice straw and sawdust) by cellulase activity of *A. agilis* in NFMM broth.

**Table 1:** Phenotypic characteristics of *A. agilis*.

Characteristics	
TSI	**+**
Motility	**+**
Citrate utilization	**+**
Indole	**+**
Methyl Red	**+**
Voges Proskauer (VP)	**-**
Starch hydrolysis	**+**
Gelatin liquification	**+**
Enzyme Production	
Catalase	**+**
Urease	**+**
Cellulase	**+**
Gowth at/ with	
NaCl, 3%-12%	**+**
Utilization of sugar Glucose, sucrose, fructose, arabinose, cellulose, CMC, mannitol	**+**
Susceptible to	
Kanamycin (30µg)	**-**
Streptomycin (25µg)	**-**
Resistance to	
Ampicillin (30µg)	**+**
Chloramphenicol (10 µg)	**+**

TSI – Triple Sugar Iron test, Voges Proskauer- VP test

**Table 2:** Ammonia concentrations of *A. agilis* in NFMM broth from utilization of various carbon sources

Carbon Sources	*Azomonas agilis*	
	Estimated Ammonium concentration by Ammonium test kit (ppm)	Ammonium concentration by Indophenol Method (ppm)
Fructose	> 3	11.893
Glucose	2-3	4.691
Sucrose	> 3	14.665
Mannitol	3	7.518
Cellulose	2	3.109
CMC	1	1.914
Arabinose	-	0.326
